# Triterpenes from *Stachyurus himalaicus* var. *himalaicus* Hook. f. et Thoms. ex Benth

**DOI:** 10.3390/molecules15042096

**Published:** 2010-03-24

**Authors:** Yun-Song Wang, Rong Huang, Ning-Zhong Li, Hong-Yi Xu, Jing-Hua Yang

**Affiliations:** 1 Key Laboratory of Medicinal Chemistry for Natural Resource, Ministry of Education, School of Chemical Science and Technology, Yunnan University, Kunming 650091, China; E-Mail: wangys@ynu.edu.cn (Y.-S.W.); 2 Xin Feng Hospital of Xi-Shang District, Kunming 650100, China; E-Mail: liningzhong@hotmail.com (N.Z.L.); 3 Department of Public Security of Yunnan Province, Kunming 650031, China; E-Mail: hongyix@sina.com (H-Y.X.)

**Keywords:** stachyuraceae, *Stachyurus himalaicus* var. *himalaicus* Hook. f. et Thoms. Ex Benth, triterpenes, stachlic acid C

## Abstract

Four triterpenes **1**–**4**, including a new naturally occurring oleanane-type triterpene **1**, were isolated by a multi-step chromatography procedure from the leaves and twigs of *Stachyurus himalaicus* var. *himalaicus* Hook. f. et Thoms.ex Benth. The structures of the compounds were elucidated by spectroscopic methods, including HRESIMS, ^1^H- NMR, ^13^C-NMR, DEPT, HMQC, HMBC and NOESY spectra. All the isolated compounds were evaluated for their *in vitro* cytotoxic activities against human Hela cell line.

## 1. Introduction

*Stachyurus*, the only genus in the Stachyuraceae family, consists of 10 species distributed in Eastern Asia [1]. Early studies regarding the chemical constituents of *Stachyurus* revealed the presence of ellagitannins, hydrolysable tannins and complex tannins [2,3,4]. *Stachyurus himalaicus* var. *himalaicus* Hook. f. et Thoms. ex Benth is known as “Tong-Cao” in Chinese folklore, and has been used for a long time as a galactopoietic, diuretic, and for the treatment of dropsy and gonorrhea [1]. Our preliminary pharmacological study on this plant showed the EtOH extract to have cytotoxic activity with an IC_50_ value of 23.5 μg/mL against the human Hela cell line. Further bioassay guided study revealed that the EtOAc extract was active against the Hela cell line with an IC_50_ value of 7.5 μg/mL. Our previous research on this plant resulted in the isolation of two new polyoxygenated triterpenes and steroids [5,6]. We herein present the structural elucidation of the newly isolated triterpenes and their cytotoxic activities.

## 2. Results and Discussion

**Figure 1:**
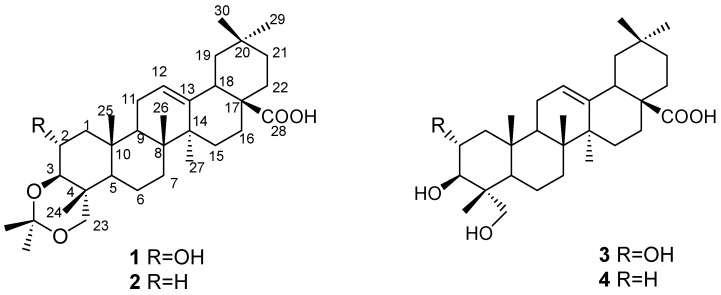
The structures of compounds **1–4** from *Stachyurus himalaicus* var. *Himalaicus*.

The powdered twigs and leaves of *S. himalaicus* var. *himalaicus* were repeatedly extracted with 90% EtOH at room temperature to afford an EtOH extract, which was suspended in H_2_O, and sequentially partitioned with petroleum ether, EtOAc, and *n*-BuOH. The EtOAc extract was subjected to silica gel column chromatographic separation to afford a total of 19 fractions (I-XIX). Further separation of fractions XIII, XIV, and XV through silica gel and subsequent Sephadex LH-20 column chromatography yielded compounds **1–4** (Figure 1).

Compound **1** was isolated as a white amorphous powder. The molecular formula C_33_H_52_O_5_ was deduced from the negative HR-ESI-MS ([M-1]- at *m/z* 527.3741, calcd. 527.3736), corresponding to eight degrees of unsaturation. The IR spectrum displayed absorption bands for hydroxyl (3,433 cm^−1^) and carbonyl (1697 cm^−1^) groups. Compound **1** gave a positive result in the Liebermann-Burchard test. The mass spectrum of **1** produced important fragments at *m/z* 248 and 203, which arose from retro Diels-Alder cleavage around ring C. This is a characteristic fragmentation pattern for an olean-12-ene triterpene [8,9]. Analysis of the NMR spectra indicated the presence of six tertiary methyl groups, an olefinic double bond [δ 5.28 (brs, H-12), δ 122.6(d, C-12) and 143.9 (s, C-13)], a carbonyl carbon at δ 183.5 (s, C-28), and a β-18 proton at δ 2.82 (dd, *J* = 3.7, 13.7 Hz), that are typical signals of an olean-12-en skeleton (Table 1).

Besides, its ^13^C-NMR and DEPT spectra showed three oxygen-bearing carbon signals at δ 65.6 (d, C-2), 82.4 (d, C-3) and 73.0 (t, C-23) (Table 1).

**Figure 2:**
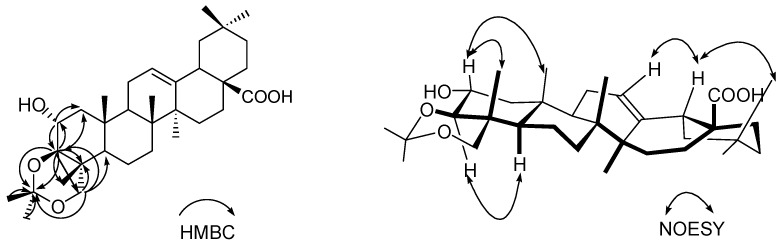
The key HMBC(H→C)and NOESY(H→H)correlations of **1**.

Comparison of the ^1^H and ^13^C-NMR data of **1** with those of compound **3** suggested that **1** was also an olean-12-en-28-oic acid bearing 2α-, 3β- and 23-oxy groups. In comparison with **3**, **1** has two additional methyls [δ 19.8 (q), 30.0 (q)] and one quaternary carbon (δ 100.0, s), which suggests an additional isopropylidene moiety [10]. The isopropylidene was assigned to the oxygenated carbon atoms at C-3 and C-23 to form a six-membered 1,3-dioxane moiety due to the downfield shifts of the C-3 and C-23 signals in comparison with those of **3**. This was confirmed by the presence of HMBC correlations of H-3 (δ 3.32) to C-1 (δ 46.2, t), C-2 (65.6, d), C-23 (73.0, t), C-4 (37.3, s), C-24 (13.8, q) and the quaternary oxygenated carbon at δ100.0 (s), and H-23 (δ3.49 and 3.46) to C-24 (δ13.8, q), C-3 (82.4, d), C-2 (65.6, d), C-4 (37.3, s) and the quarternary carton at δ100.0 (s) Thus, the structure of **1** was established as 3β, 23-*O*-isopropylidenyl-2α,3β,23-trihydroxyolean-12-en-28-oic acid, named stachlic acid C (Figure 1), by the combined analysis of the ^1^H- and ^13^C-NMR (Table 1), COSY, HMBC, and NOESY spectral data (Figure 2). Compound **1** was previously obtained by synthesis from arjunolic acid with treatment of acetone and anhydrous cupric sulfate [11]. But as a natural product, compound **1** was isolated here for the first time from Nature.

Compound **2** has been reported previously from the roots of *Caulophyllum robustum* Maxim [12], but its spectral data are reported here for the first time. Compounds **3** and **4** were identified as arjunolic acid and hederagenin, respectively, by a combination of spectroscopic methods and comparisons with the literature data [13,14]. Compounds **1** and **2** possessed an isopropylidene moiety and are monoacetonides of **3** and **4**, respectively. Triterpenes and ecdysteroids with an isopropylidene moiety were previously isolated from the same plant [5,6]. No acetone was used during our chromatographic operation procedure, so the isolates should be naturally occurring triterpenoids. Compounds **2**–**4** were isolated for the first time from *S. himalaicus* var. *himalaicus*. All isolates were screened in a cytotoxicity assay. Among them, the compounds **2**, **3** and **4** were found to have weak cytotoxic activity against human Hela cell line *in vitro*, but **1** was not cytotoxic against human Hela cell line. The IC50 values were summarized in Table 2.

## 3. Experimental

### 3.1. General

Commercial silica-gel plates (Qing Dao Marine Chemical Group Co.) were used for TLC analyses. Melting point was measured on a XRC-1 micro-melting point apparatus and uncorrected. UV/VIS Spectra were measured on a Shimadzu UV-2401PC spectrophotometer; λ_max_ in nm. IR spectra were obtained on a Bio-Rad FTS-135 infrared spectrophotometer, ν_max_ in cm-1. 1H- and 13C- NMR as well as 2D-NMR spectra were recorded on a Bruker DRX-300 or a DRX-500 spectrometer with TMS as internal standard, coupling constant *J* in Hz. MS spectra were performed on a VG Autospec-3000 mass spectrometer.

### 3.2. Plant material

The leaves and twigs of *S. himalaicus* var. *himalaicus* Hook. f. et Thoms. ex Benth were collected in Wenshan County of Yunnan Province, P. R. China, in May 2003 and identified by Professor Zhi-Hao Hu of the Department of Botany, Yunnan University, Kunming, China. A voucher specimen (200305) is deposited in Key Laboratory of Medicinal Chemistry for Natural Resource, Ministry of Education, Yunnan University.

### 3.3. Extraction and isolation

The powdered leaves and twigs of *S. himalaicus* var. *himalaicus* (33 kg) were repeatedly extracted with 90% EtOH (150 L) at room temperature. The extract was then concentrated under reduced pressure to give a brown syrup (2.5 kg), which was sequentially partitioned between H_2_O and petroleum ether (PE), EtOAc and *n*-BuOH to give corresponding fractions 180 g, 700 g and 350 g, respectively. The EtOAc extract (700 g) was subjected to silica gel column chromatography eluting successively with PE-EtOAc (10:1–1:1), EtOAc-MeOH (10:1–1:1) and MeOH to afford 19 fractions (I-XIX). Fraction XIII was purified by column chromatography over a silica gel column (PE/EtOAC 3:1 and 1:1), and then a Sephadex LH-20 column (MeOH) to give **2** (10 mg); fraction XIV was subjected to column chromatographic separation over a silica gel column by gradient elution using CHCl3-EtOAc, and then a Sephadex LH-20 column (using MeOH as eluent) to yield compound **1** (12 mg). Fraction XV was re-chromatographed on a silica gel column, and eluted with CHCl3 containing increasing amounts of MeOH, and then on a Sephadex LH-20 column (using MeOH as eluent) to give compounds **3** (40 mg) and **4** (28 mg).

### 3.4. Spectral data

*Stachlic acid C (3β,23-O-isopropylidenyl-2α, 3β, 23-trihydroxyolean-12-en-28-oic acid*, (**1**). White amorphous powder; ${\left[\alpha \right]}_{D}^{18.1}$: 30.4 (*c* 0.66; CHCl_3_). IR ν _max_ (KBr) cm^−1^: 3433, 2924, 2853, 1697, 1069; ^1^H-NMR (500 MHz, δ ppm, CDCl_3_) and ^13^C-NMR (125 MHz): see Table 1; HRESIMS *m/z* 527.3741 [M-1]- (calcd. for C_33_H_52_O_5_, 527.3736); FAB+MS *m/z* 529 [M+H]+, 471, 453, 407, 248.

*3β,23-O-isopropylidenyl-3β,23-dihydroxyolean-12-en-28-oic acid* (**2**). Colorless needles, m.p. 285–286 °C; ${\left[\alpha \right]}_{D}^{17.9}$: −13.1 (c 0.54; CHCl_3_). IR ν_max_ (KBr) cm^−1^: 3441, 2930, 2854, 1631, 1696, 1067; ^1^H-NMR (500 MHz, δ ppm, CDCl_3_) and ^13^C-NMR (125 MHz): see Table 1; HRESIMS *m/z* 511.3780 [M-1]- (calcd for C_33_H_52_O_4_, 511.3787); FAB-MS *m/z* 511 [M-H]-.

### 3.5. Cytotoxic activity

Hela (human carcinoma of the cervix) cell line were grown as a monolayer in Dulbecco’s modified eagle’s medium, DMEM (Gibco), supplemented with 10% newborn calf serum (Gibco) and 1% of penicillin-streptomycin mixture (10,000 UI/mL). The cells were maintained at 37 °C in 5% CO_2_ and 90% humidity. The cytotoxic activity was assessed using colorimetric MTT reduction assay [7]. Briefly, 5000 Hela cells per well were seeded in DMEM (100 μL) in 96-well microculture plates for 24 h. After 24 h adaptation, medium (100 μL) containing various drug concentrations were added to each well, while control cells received fresh medium containing analogous DMSO concentrations. Each concentration was tested in at least eight wells. After 72 h incubation, the medium was replaced by DMEM medium (100 μL, without serum) containing MTT solution (10 μL, 3 mg/mL in PBS). After 45 min in the incubator, the medium was removed and DMSO (100 μL) was added to each well. The plates were shaked and optical densities were recorded at 550 nm. Camptothecin (Sigma) was used as positive control. The percentage viability was plotted against the compound concentrations and the 50% cell viability (IC50) was calculated from the curve. All the experiments were repeated three times. Results were expressed as mean of IC50 values (μM) ±SEM.

## 4. Conclusions

Previous phytochemical studies had reported the presence of tannins from the genus *Stachyurus* [2,3,4]. However, very little is known regarding the chemical constituents and biological properties of the aerial part of *S. himalaicus* var. *himalaicus*. Our previous research on this plant resulted in the isolation of two new polyoxygenated triterpenes [5]. In the present study, a new naturally occurring oleanane-type triterpene **1**, named stachlic acid C, was isolated from *S. himalaicus* var. *himalaicus* along with three known triterpenes **2**, **3** and **4**. Compounds **2**–**4** were isolated for the first time from this plant. Triterpenes **1** and **2** possess an isopropylidene moiety in the molecule and the compounds **2**, **3** and **4** were found to have weak cytotoxic activity against human Hela cell line *in vitro*.

## Figures and Tables

**Table 1:** The ^1^H- and ^13^C-NMR spectra data for compounds **1**–**2** (δ in ppm, *J* in Hz ) ^a^.

Atom No.	1		2	
	*δ*_H_	*δ*_C_	*δ*_H_	*δ*_C_
1	2.05, 0.98 (each 1H, m)	46.8 (t)	1.67, 1.01 (each 1H, m)	38.9 (t)
2	3.78 (1H, ddd, 4.5, 4, 9.7)	65.6 (d)	1.61, 1.40 (each 1H, m)	23.4 (t)^b^
3	3.32 (1H, d, 9.6)	82.4 (d)	3.49 (1H, dd, 3.6, 9.5)	77.7 (d)
4	/	37.3 (s)	/	36.9 (s)
5	0.88 (1H, m)	51.8 (d)	0.78 (1H, m)	51.6 (d)
6	1.40, 0.86 (each1H, m)	17.9 (t)	1.38, 1.22 (each 1H, m)	17.8 (t)
7	1.42, 1.26 (each 1H, m)	32.4 (t)	1.41, 1.25 (each 1H, m)	32.3 (t)
8	/	39.7 (s)	/	39.6 (s)
9	1.70 (1H, m)	48.0 (d)	1.58 (1H, m)	47.8 (d)
10	/	38.5 (s)	/	37.4 (s)
11	1.94, 1.80 (each 1H, m)	23.2 (t)^c^	1.90, 1.78 (each 1H, m)	23.6 (t)^b^
12	5.28 (1H, s)	122.6 (d)	5.27 (1H, s)	122.6 (d)
13	/	143.9 (s)	/	143.7 (s)
14	/	42.0 (s)	/	41.0 (s)
15	1.67, 1.04 (each 1H, m)	28.0 (t)	1.71, 1.06 (each 1H, m)	27.8 (t)
16	1.99, 1.61 (each 1H, m)	23.6 (t)^c^	1.98, 1.41 (each 1H, m)	23.0 (t)
17	/	46.6 (s)	/	46.6 (s)
18	2.82 (1H, dd, 3.7, 13.7)	41.3 (d)	2.82 (1H, dd, 3.7, 13.3)	41.7 (d)
19	1.58, 1.14 (each 1H, m)	46.2 (t)	1.63, 1.16 (each 1H, m)	46.0 (t)
20	/	31.0 (s)	/	30.8 (s)
21	1.34, 1.20 (each 1H, m)	34.1 (t)	1.34, 1.22 (each 1H, m)	33.9 (t)
22	1.77, 1.56 (each 1H, m)	32.8 (t)	1.76, 1.56 (each 1H, m)	32.6 (t)
23	3.49, 3.46 (each 1H, d, 10.7)	73.0 (t)	3.44, 3.52 (each 1H, d, 10.7)	72.8 (t)
24	1.06 (3H, s)	13.8 (q)	1.04 (3H, s)	12.6 (q)
25	1.02 (3H, s)	18.1 (q)	0.96 (3H, s)	16.6 (q)
26	0.73 (3H, s)	17.4 (q)	0.72 (3H, s)	17.2 (q)
27	1.14 (3H, s)	26.3 (q)	1.14 (3H, s)	26.1 (q)
28	/	183.5 (s)	/	184.4 (s)
29	0.91 (3H, s)	33.4 (q)	0.90 (3H, s)	33.2 (q)
30	0.93 (3H, s)	23.9 (q)	0.92 (3H, s)	23.7 (q)
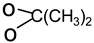	1.45, 1.44 (each 3H, s)	100.0 (s) 19.8 (q) 30.0 (q)	1.45, 1.42 (each 3H, s)	99.1 (s) 19.5 (q) 30.0 (q)

^a^ **1** and **2** were recorded in CDCl_3_; ^b,c^ Assignment may be interchargeable.

**Table 2:** Cytotoxicity data on Hela Cells for compounds **1**–**4**^a^.

Compound	IC_50_ (μM)
1	> 142
2	29.3 ± 3.7
3	49.8 ± 4.3
4	19.3 ± 6.8
Camptothecine	0.5

^a^ Results are expressed as mean of IC_50_ values (μM) ± SEM.
